# Functional drug-delivery hydrogels for oral and maxillofacial wound healing

**DOI:** 10.3389/fbioe.2023.1241660

**Published:** 2023-08-03

**Authors:** Ming Hao, Dongxu Wang, Mengna Duan, Shaoning Kan, Shuangji Li, Han Wu, Jingcheng Xiang, Weiwei Liu

**Affiliations:** ^1^ Department of Oral and Maxillofacial Surgery, Hospital of Stomatology, Jilin University, Changchun, China; ^2^ Jilin Provincial Key Laboratory of Tooth Development and Bone Remodeling, Hospital of Stomatology, Jilin University, Changchun, China; ^3^ Laboratory Animal Center, College of Animal Science, Jilin University, Changchun, China; ^4^ Department of Prosthodontics, Hospital of Stomatology, Jilin University, Changchun, China

**Keywords:** functional hydrogel, skin repair, wound healing, oral and maxillofacial, drug delivery

## Abstract

The repair process for oral and maxillofacial injuries involves hemostasis, inflammation, proliferation, and remodeling. Injury repair involves a variety of cells, including platelets, immune cells, fibroblasts, and various cytokines. Rapid and adequate healing of oral and maxillofacial trauma is a major concern to patients. Functional drug-delivery hydrogels play an active role in promoting wound healing and have shown unique advantages in wound dressings. Functional hydrogels promote wound healing through their adhesive, anti-inflammatory, antioxidant, antibacterial, hemostatic, angiogenic, and re-epithelialization-promoting properties, effectively sealing wounds and reducing inflammation. In addition, functional hydrogels can respond to changes in temperature, light, magnetic fields, pH, and reactive oxygen species to release drugs, enabling precise treatment. Furthermore, hydrogels can deliver various cargos that promote healing, including nucleic acids, cytokines, small-molecule drugs, stem cells, exosomes, and nanomaterials. Therefore, functional drug-delivery hydrogels have a positive impact on the healing of oral and maxillofacial injuries. This review describes the oral mucosal structure and healing process and summarizes the currently available responsive hydrogels used to promote wound healing.

## 1 Introduction

The oral and maxillofacial region is involved in important physiological functions, such as respiration, mastication, articulation, esthetic maintenance, and facial expression production, and involves complex anatomical structures, including skin, mucosa, periodontal tissue, teeth, and salivary glands (Madani et al., 2014; van Veen et al., 2019; Zhai et al., 2019; Matichescu et al., 2020; Ulaganathan et al., 2020; Chung et al., 2021; Hatcher, 2022; Hong, 2023). Thus, the healing of oral and maxillofacial wounds is of great importance to maintaining the integrity of its anatomical structure and function (Ha et al., 2023). Wounds are defined as the disruption of anatomical structures accompanied by loss of continuity of function, which can be repaired by a unique, timely, and spatially coordinated wound healing process (Chhabra et al., 2017; Li et al., 2022a). Wounds that fail to heal can, even indirectly, lead to death (Zeng et al., 2018). The characteristics of non-healing wounds include long-term or excessive inflammation, persistent infection, microbial biofilm formation, and the incapacity of dermal or epidermal cells to react to repairing stimuli (Frykberg and Banks, 2015). Non-healing wounds may lead to permanent damage to the normal function and appearance of the oral and maxillofacial region.

Hydrogels have been found to effectively improve wound healing and functional recovery (Zhang et al., 2020). Hydrogels have unique advantages in wound dressings due to their excellent biological, physicochemical, and mechanical properties (Alven and Aderibigbe, 2020), including excellent biocompatibility (Graca et al., 2020), a biological barrier effect blocking harmful factors, such as bacteria, from entering the wound (Norahan et al., 2023), and the ability to deliver cargos that promote wound healing in response to environmental changes (Dimatteo et al., 2018). It is a great concern that antibiotic overuse has led to the development of drug-resistant bacterial strains. Therefore, a variety of antimicrobial hydrogels have recently been synthesized to effectively avoid development of drug-resistant bacteria (Farazin et al., 2023). Thus, hydrogels have widespread applications in the field of wound healing, and various functional hydrogels have been developed with adhesive, antioxidant, antibacterial, hemostatic, and tissue regenerative properties (Liang et al., 2021). Oral and maxillofacial wounds require hydrogels with good adhesion to provide wound protection; however, their degradation time and on-demand removal need to be considered to prevent rapid degradation exposing the wound or leading to secondary injury when removing the hydrogel (Ding et al., 2022; Lv et al., 2023). Moreover, hydrogels can respond to stimuli, such as temperature, light, magnetic fields, pH, and reactive oxygen species (ROS), to release active cargos (for example, nucleic acids, growth factors, small-molecule drugs, stem cells, exosomes, and nanomaterials) (Zhang et al., 2023).

This review summarizes the oral and maxillofacial wound healing process as well as the factors that influence healing. Functional and responsive hydrogels which are effective treatments to promote wound healing are also introduced in categories.

## 2 The process of oral and maxillofacial wound healing

### 2.1 Epithelium of skin and oral mucosa

The skin and mucosa of the oral and maxillofacial region, as barriers and protective organs of the body, are important for the maintenance of water–electrolyte balance and protection of internal tissues and organs from microbial infection and external friction, which would affect their function and esthetics (Wang et al., 2017; Park et al., 2018).

**FIGURE 1:**
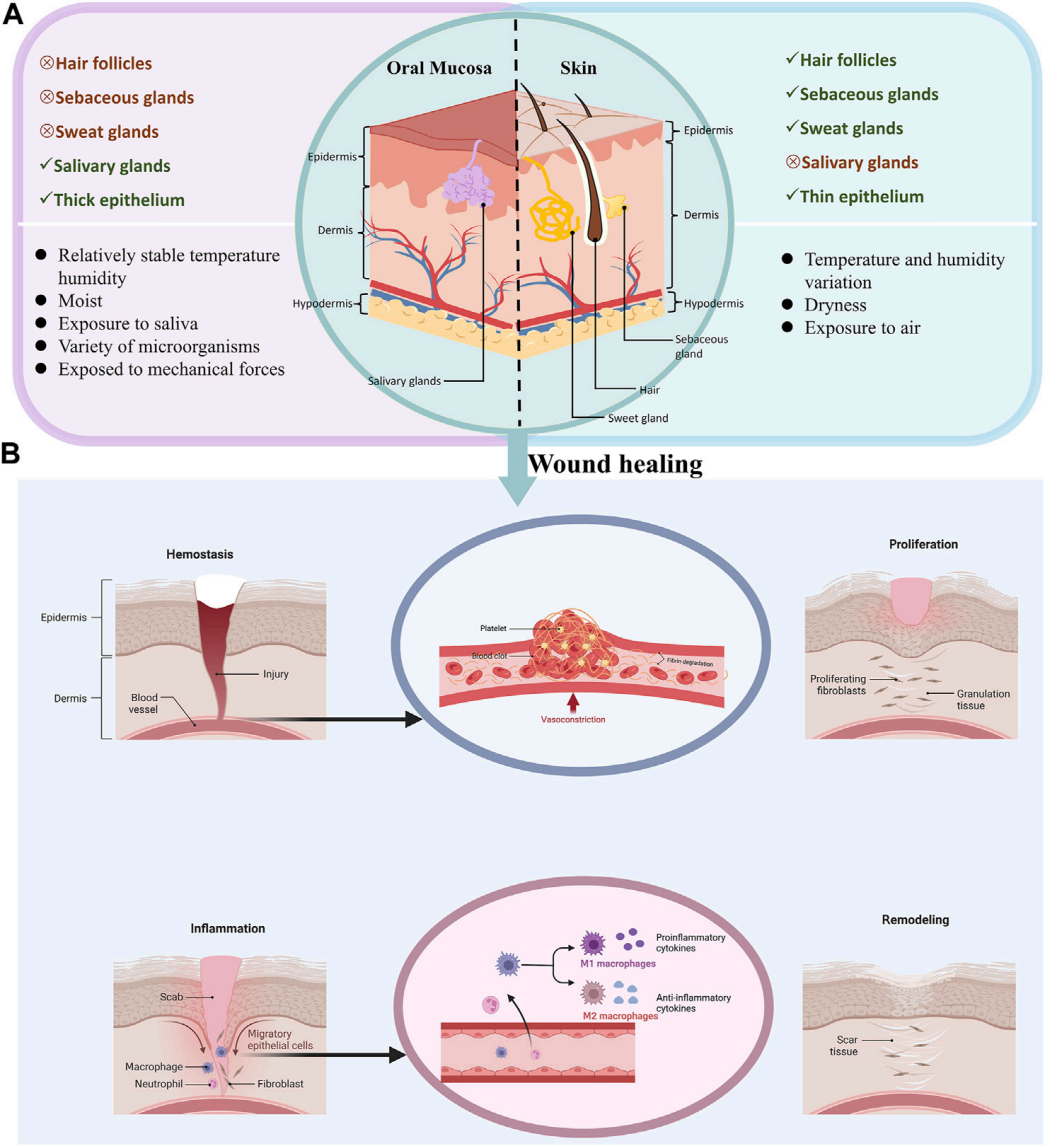
Differences in oral mucosa and skin anatomy and healing process. **(A)**, The differences between oral mucosa and skin anatomy. **(B)**, The healing process in oral mucosa and skin.

The oral mucosa and skin epithelium consist of superficial epithelium and an underlying basement membrane (Toma et al., 2021). The skin consists of a keratinized epidermal layer, dermis, and subcutaneous layer (Glim et al., 2013). The epithelium of the skin contains hair follicles and pores, which serve as a transmembrane absorption pathway providing a therapeutic modality (Glim et al., 2013; Barbero and Frasch, 2017). However, the oral mucosa consists of non-keratinized epithelium, a loose extracellular matrix (ECM), and salivary glands (Groeger and Meyle, 2019; Liu et al., 2019), and may vary according to the different environments in which the skin and mucous membranes are located. Furthermore, the oral mucosa is in a complex, warm, and moist environment with a diverse microbial presence and is subject to friction from mastication (Mark Welch et al., 2020). In contrast, the skin is exposed to changes in air, temperature, and humidity (Waasdorp et al., 2021). Moreover, studies have shown that the oral mucosa heals faster than the skin and that fewer scars are formed, likely due to the lower response to inflammation and the presence of saliva in the oral environment (Brand et al., 2014). Recent research has indicated that saliva contains various substances, such as antimicrobial peptides, histatins, and mucins, that can promote wound healing by interacting with fibroblasts, keratinocytes, and growth factors (Torres et al., 2018). In addition, oral mucosa has a rich blood supply and contains numerous capillaries, which is beneficial for wound healing (Han et al., 2018). There are significant differences in tissue structure and physiological characteristics between oral mucosa and skin that lead to variations in the healing process (Figure 1A; Table 1).

**TABLE 1:** The difference in structure, surroundings and healing processes between oral mucosa and skin.

	Oral mucosa	Skin	References
Structures	Epithelial and lamina propria, part of the oral mucosa has submucosa	Epidermis, dermis and subcutaneous tissue	Glim et al. (2013)
	Keratinization/Incomplete keratinization	Keratinization	Qin et al. (2017)
	Salivary glands	Hair follicles, sebaceous glands, sweat glands	Glim et al. (2013)
	Thicker epithelium	Relatively thin epithelium	Glim et al. (2013)
	High proliferation rate of basal cells	Low proliferation rate of basal cells	Gibbs and Ponec (2000)
	Relatively high degree of vascularization	Relatively low degree of vascularization	Brand et al. (2014)
Surroundings	Epidermal moistening	Epidermal dryness	Brand et al. (2014)
	Exposure to saliva	Exposure to air	Brand et al. (2014)
	Subjected to chewing force and tension, continuously exposed to bacteria		Dutzan et al. (2017)
Healing process	The oral mucosa has less neovascularization than the skin		Szpaderska et al. (2005)
		Fibroblasts are more responsive to stimulus	Mak et al. (2009)
	Lower inflammation levels		Szpaderska et al. (2003), Chen et al. (2010)
	Saliva contains high levels of healing-promoting histone and growth factors		Brand et al. (2014)
	Oral keratinocytes have a proliferative capacity greater than that of skin keratinocytes		Turabelidze et al. (2014)
	Oral wounds exhibit rapid re-epithelialization		Schrementi et al. (2008)

### 2.2 Oral and maxillofacial wound healing

Following traumatic injuries, such as car accidents, knife wounds, cuts, and surgery, wounds heal through hemostasis, inflammation, proliferation, and remodeling, with the involvement of multiple cells and cytokines in the oral and maxillofacial region. The process of healing is described below (Figure 1B).

#### 2.2.1 Hemostasis

Upon skin injury and damage to blood vessels, bleeding occurs immediately. Hemostasis subsequently occurs with the activation of platelets, thus initiating the hemostasis cascade (Burzynski et al., 2019). Subsequently, platelets produce a number of biologically active substances, including proteases, growth factors and cytokines such as epidermal growth factor (EGF), insulin-like growth factor (IGF), platelet-derived growth factor (PDGF), and transforming growth factor-β (TGFβ) (Politis et al., 2016; Scopelliti et al., 2022). Moreover, blood vessels constrict and platelets aggregate to form a platelet plug (Scopelliti et al., 2022).

#### 2.2.2 Inflammation

Inflammation is caused by fibrin clot formation and aggregated platelet degranulation (Pazyar et al., 2014). In the early stages, neutrophils migrate to the site of injury and secrete enzymes, such as matrix metalloproteinases (MMPs), to remove the damaged ECM. Neutrophils then initiate cytokine and growth factor production to recruit monocytes (Velnar et al., 2009). Monocytes migrate to the wound and differentiate into macrophages (Velnar et al., 2009). Macrophages secrete cytokines, including interleukin-1 (IL-1), interleukin-6 (IL-6), fibroblast growth factor (FGF), PDGF, EGF, tumor necrosis factor (TNF), and TGFβ, which coordinate the migration of keratinocytes and fibroblasts to the wound (Sindrilaru and Scharffetter-Kochanek, 2013). Macrophages are characterized by their ability to respond to environmental changes and can differentiate into two distinct subpopulations: pro-inflammatory M1 macrophages and anti-inflammatory M2 macrophages (Gordon and Taylor, 2005; Smigiel and Parks, 2018). M2 macrophages contribute to the upregulation of endogenous anti-inflammatory cytokines and the downregulation of previously secreted pro-inflammatory cytokines in the vicinity of the wound (Krzyszczyk et al., 2018). Indeed, abnormal macrophage polarization has ability to delay wound healing or lead to chronic wound formation (Louiselle et al., 2021). Nevertheless, excessive infiltration of inflammatory cells, including neutrophils and macrophages, inhibits wound healing (Eming et al., 2007).

#### 2.2.3 Proliferation

At this stage, the matrix formed by platelets is replaced by vascularized granulation tissue and re-epithelialization occurs, leading to wound closure and reestablishment of the epithelial barrier (An et al., 2021). Re-epithelialization proceeds through proliferation and migration of keratinocytes, which is influenced by nitric oxide, EGF, IGF1, keratinocyte growth factor (KGF), and nerve growth factor (Landen et al., 2016). Reconstruction of the existing vascular network and formation of new vessels are the hallmarks of successful wound healing (Toma et al., 2021). Newly formed blood vessels provide nutrients and oxygen to the tissues (Li et al., 2007). Furthermore, studies have shown that several growth factors participate in angiogenesis, including vascular endothelial growth factor (VEGF), angiopoietin, FGF, and TGFβ (Li et al., 2003), and that the production of a temporary matrix in the granulation tissue is facilitated by the involvement of fibroblasts (Darby et al., 2014). Furthermore, fibroblasts differentiate into α-smooth muscle actin-expressing myofibroblasts, which initiate the wound closure process (Zhang et al., 2020b). Nevertheless, hyperactivation of fibroblasts and myofibroblasts is associated with abnormal scar formation (Zhang et al., 2020b). Moreover, macrophages are also involved in granulation tissue formation, secreting a variety of growth factors (FGF, TGFβ, EGF and PDGF) (Barrientos et al., 2008).

#### 2.2.4 Remodeling

The final stage of healing is remodeling, which is intended to remove unnecessary vessels, fibroblasts, and inflammatory cells (Nourian Dehkordi et al., 2019). Wound closure mainly occurs through granulation and re-epithelialization. However, granulation tissue is a temporary matrix. Indeed, an important feature of wound remodeling is the change in composition of the ECM. Thus, the structure of the ECM fibrillar network undergoes organization and alignment (Li et al., 2007). Fibroblasts remodel the ECM by secreting ECM-degrading enzymes. Active proteases, especially MMPs, are involved in wound healing by controlling the synthesis of new collagen and degradation of old collagen (Visse and Nagase, 2003). The previous, temporary ECM then transitions from a loose network of fibronectin tissue to a larger, denser collagen bundle. Type III collagen is gradually transformed into type I collagen (Finnerty et al., 2016). Collagen alignment also tends to be consistent, thus increasing the strength of new tissue. Skin appendages, such as hair, sweat glands, and sebaceous glands, may regenerate (Feng et al., 2019).

### 2.3 The formation of hard-to-heal and poorly healed wounds

When a stage of wound healing is compromised, it can result in chronic wounds. The main features of chronic wounds include a prolonged inflammatory phase, excessive infiltration of neutrophils, persistent infection and high levels of MMPs. Fibroblasts in chronic wounds and ulcers are phenotypically different and have a reduced migratory capacity compared to those in acute wounds (Brumberg et al., 2021). The dysregulation of key pro-inflammatory cytokines, such as IL-1β and TNF, prolongs the inflammatory phase and delays healing. IL-1β and TNF are increased in chronic wounds, which have been shown to lead to elevated MMP levels and excessive degradation of the local ECM, impairing cellular migration (Hesketh et al., 2017; Krzyszczyk et al., 2018). It has been shown that MMPs, such as collagenase and gelatinase A and B, are elevated in the fluid of chronic wounds compared to that in acute wounds (Eming et al., 2010). Additionally, inadequate angiogenesis is considered a potential cause of chronic wounds. This is related to the deprivation of pro-angiogenic factors, such as members of the VEGF family, by proteolytic degradation, followed by subsequent interference with their bioactivity in the microenvironment of chronic wounds (Lauer et al., 2000). The formation of chronic wounds involves multiple cellular and signaling pathways. Several molecular markers downstream of the Wnt signaling pathway, such as activation of the β-catenin/c-myc pathway, promotes healing of damaged tissue by inhibiting the migration of keratinocytes and altering their differentiation to suppress keratinization (Stojadinovic et al., 2005; Stojadinovic et al., 2014). The EGF receptor is inhibited in keratinocytes at the non-healing edge (Brem et al., 2007). In addition, TGFβ signaling is suppressed in chronic wounds through the downregulation of TGFβ receptors and attenuation of Smad signaling (Pastar et al., 2010). Therefore, such cell signaling can serve as a target for rapid wound healing of chronic wounds.

## 3 Functional hydrogels for wound healing

**FIGURE 2:**
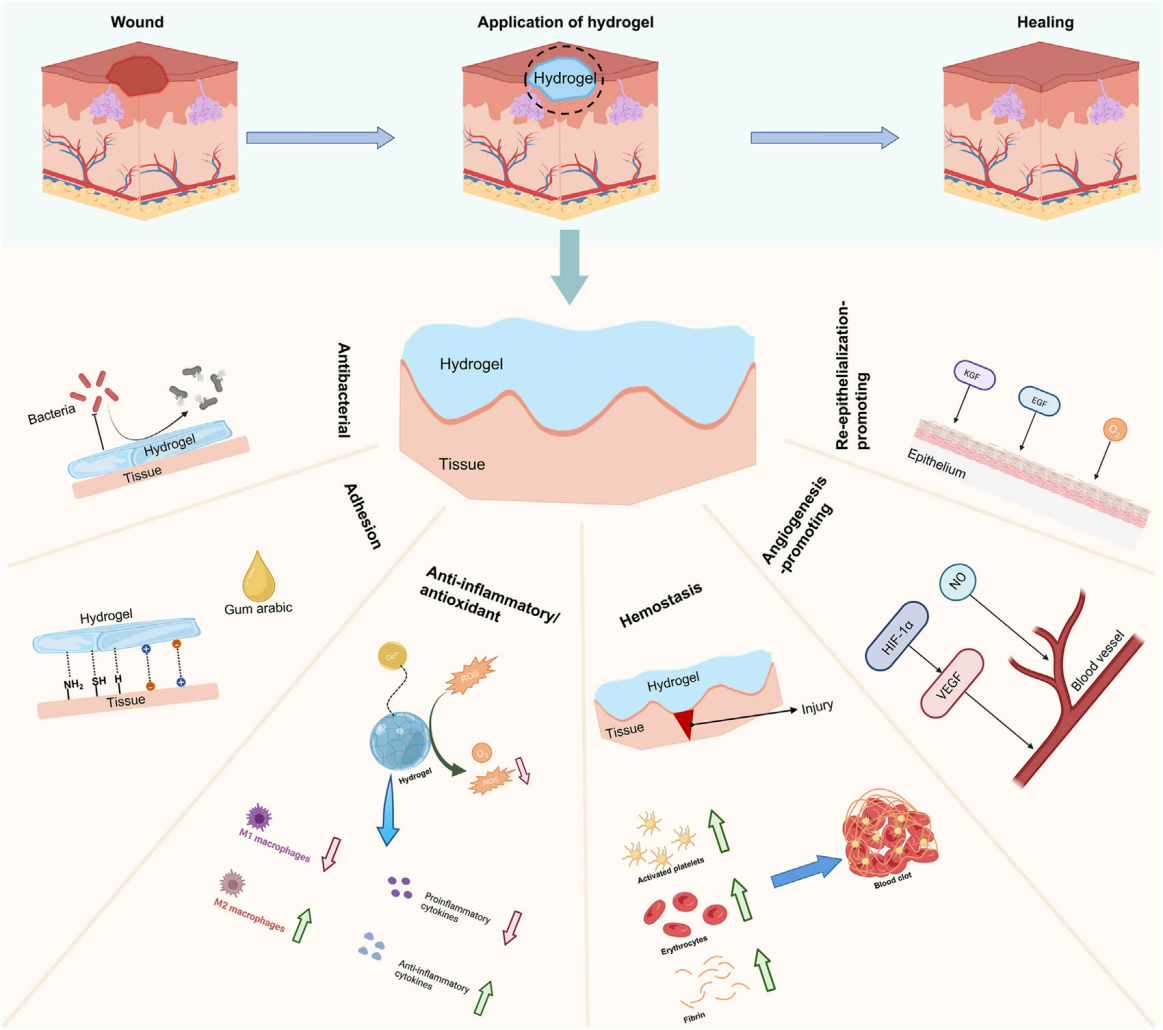
Classification of functional hydrogels.

Traditional wound dressings, including gauze and bandages, are commonly used for dry and clean wounds (Brumberg et al., 2021). Compared to conventional wound dressings, hydrogels have great advantages and application prospects due to their porous structure, biodegradability, and drug-carrying and sustained release capacity (Kamoun et al., 2017; Bai et al., 2020b). Hydrogels can be designed by modulating their properties to match the type of wound and trauma process, with the development of hydrogels with adhesive, anti-inflammatory/antioxidant, antibacterial, hemostatic, angiogenesis-promoting, and re-epithelialization-promoting properties (Figure 2).

### 3.1 Adhesive hydrogels

The oral mucosa is affected by functional movements and constant flushing when chewing food and processing saliva, making it difficult for drugs and dressings to stay in a moist environment (Wu et al., 2022a). Therefore, hydrogels that can remain adherent in wet environments have been designed inspired by the superb adhesion of mussels in humid environments. Hydrogels with better adhesion properties than commercial dressings were synthesized by photocrosslinking polyethylene glycol (PEG) monomethyl ether-modified glycidyl methacrylate-functionalized chitosan (CS), double bond-modified dopamine, and zinc ions (Yang et al., 2022). These hydrogels remained adherent to the wound for more than a few hours in a humid environment in the mouth. A light-responsive hydrogel of cyclo-nitrobenzene-modified hyaluronic acid was designed to bind firmly to intraoral wound tissue within seconds of light exposure and was adherent for more than 24 h (Zhang et al., 2021b). Given that the skin and muscles of the oral and maxillofacial region are involved in a variety of important physiological functions, the adherent hydrogel must be tough and self-healing to resist external friction (Sun et al., 2021). A super-tough hydrogel with excellent tissue affinity, adhesion, and self-healing properties was prepared by physical and chemical double cross-linking providing firm protection for wounds (Chen et al., 2018b). Moreover, a common hydrogel prepared from gum Arabic has good adhesion to the tissue interface and does not affect the organization’s movement (Singh et al., 2017). Furthermore, the excellent bonding ability of hydrogels can enhance wound contraction and closure during the early stages of wound healing (Sun et al., 2016). Thus, adhesive hydrogels can adhere to the wound and protect it, thereby promoting healing.

### 3.2 Anti-inflammatory/antioxidant hydrogels

The inflammatory response is a phase of wound healing that is susceptible to interference from internal and external oral surroundings, resulting in excessive inflammation. Moreover, prolonged inflammation leads to the accumulation of ROS in the wound and limits the antioxidant capacity of cells to clear excess ROS (Wagener et al., 2013). Therefore, anti-inflammatory hydrogels that can eliminate ROS, and thus reduce the production of pro-inflammatory chemokines, are useful for the regulation of wound healing. Natural polysaccharides, polyphenols, and polypeptides have excellent anti-inflammatory and antioxidant abilities (Fernandes et al., 2023). Previous studies have made use of paramylon, a natural algal polysaccharide, to prepare a hydrogel that can effectively reduce wound inflammation, clear ROS, and promote rapid healing of chronic burn wounds (Lei et al., 2022). The hydrogel was synthesized utilizing the antioxidant properties of tannins, natural polyphenols, forming Fe^3+^-tannin complex-modified P(AM-AA) hydrogels (Fe-TA@P(AM-AA)). The hydrogel releases tannin, which reacts with ROS, reversing excessive infiltration of neutrophils and the imbalance between M1 and M2 macrophages, thus promoting rapid wound healing (Sun et al., 2023a). Hydrogels made from pearl peptides enhance cellular viability and resistance to oxidative stress in wound healing (Liu et al., 2022). In addition to natural ingredients, artificially synthesized metallic materials also possess antioxidant abilities. By combining thiol hyaluronic acid and bioactive silver lignin nanoparticles, a hybrid nanocomposite hydrogel was prepared with excellent antioxidant activity and chronic wound healing promotion (Perez-Rafael et al., 2021). MnO_2_ also possesses bioenzyme-like activities. Hydrobranched poly-L-lysine-modified MnO_2_ was cross-linked with hydrophilic poly(PEGMA-co-GMA-co-AAm) polymers to produce a hydrogel in which MnO_2_ showed peroxidase-like activity to decrease ROS levels and catalyze O_2_ production. This hydrogel significantly enhanced the polarization of M2 macrophages (Tu et al., 2022). Thus, hydrogels can achieve anti-inflammatory and antioxidant effects through multiple pathways.

### 3.3 Antibacterial hydrogels

The oral cavity contains a diverse range of microorganisms, including many bacteria and fungi, making oral cavity wounds easily susceptible to bacterial infections. The development of suitable biological dressings to maintain a non-sterile wound environment is of great importance for wound healing. Antibacterial hydrogels can be obtained by careful design of the components. PEG diglycidyl ether and ε-polylysine were used to synthesize antimicrobial hydrogels, in which ε-polylysine is a cationic peptide that kills bacteria and PEG diglycidyl ether prevents bacteria from adhering to the hydrogel, thus achieving an antibacterial and sterile wound environment (Yuan et al., 2021). Compared to traditional dressings, hydrogels also have better therapeutic effects on wounds that are prone to bacterial infections (Shi et al., 2020). Near-infrared (NIR)-responsive wound dressings have been developed, which can generate heat under NIR (808 nm) irradiation to kill bacteria and accelerate deep burn wound healing (Wang et al., 2022c).

The clinical application of antibiotics often leads to the development of drug resistance in bacteria. Hydrogels can easily address this; for example, hydrogels synthesized from bovine serum albumin can effectively kill methicillin-resistant *Staphylococcus aureus* (Ouyang et al., 2022). A hydrogel with excellent antimicrobial properties against extensively drug-resistant bacteria was synthesized from CS and β-glycerophosphate, promoting full wound healing of extensively drug-resistant infections (Aliakbar Ahovan et al., 2020). Therefore, through the design of hydrogel components, it is possible to gain antibacterial effects against a variety of bacteria and antibiotic-resistant strains.

### 3.4 Hemostatic hydrogels

The oral and maxillofacial areas are rich in blood vessels and highly susceptible to bleeding after trauma. Therefore, timely hemostasis is essential to prevent shock and death by bleeding. Hydrogels can promote hemostasis through physical closure and enrichment of coagulation factors (Ghobril and Grinstaff, 2015). Hydrogels for rapid hemostasis have been prepared using catechol-functionalized quaternized CS (CQCS) and dibenzaldehyde-capped PEG (DB-PEG), which has good adhesion to the wet tissue–blood interface and can cope well with bleeding by physical closure in a variety of situations (Fang et al., 2023). In addition, the hemostatic hydrogel can stop bleeding by activating platelets. Platelet activation and blood cell aggregation can be induced through the electrostatic interaction, thereby promoting hemostasis (Fan et al., 2016). Cationic CS hydrogels can interact with negatively charged blood cells to promote coagulation (Xu et al., 2013) and hemostasis can be achieved through Ca^2+^ activation of thrombin, significantly improving the hemostatic properties (Weng et al., 2022). Thus, hydrogels provide excellent hemostasis through physical sealing or through the activation of platelets and thrombin.

### 3.5 Angiogenesis-promoting hydrogels

Oral and maxillofacial healing requires blood vessels to deliver nutrients and oxygen, and impaired angiogenesis can lead to difficult-to-heal wounds. Therefore, hydrogels have been developed to have intrinsic properties promoting or stimulating angiogenesis through the loading of pro-angiogenic substances. Bioactive glass nanoparticles containing copper (BGNC) and sodium alginate (ALG) hydrogels synthesized by PEG diacrylate, bioactive glass nanoparticles containing copper, and sodium alginate were able to significantly promote angiogenesis by enhancing HIF1α and VEGF expression (Li et al., 2020). Amino acids are the basic units of proteins and have a regulatory effect on the proliferation of vascular endothelial cells during wound healing. Amino acids have been added into CS–collagen hydrogels with excellent pro-angiogenic effects (Aleem et al., 2019). These hydrogels can also have excellent angiogenesis-promoting ability by loading small-molecule drugs or bioactive components that promote angiogenesis. Desferrioxamine (DFO), an FDA-approved pro-angiogenic small molecule, and hydroxy saffron yellow A were loaded into a gelatin–CS hydrogel, which upregulated HIF1α expression and promoted angiogenesis, further promoting rapid healing of diabetic wounds (Gao et al., 2018). Aptamer–Fibrinogen hydrogels with a high affinity and specificity for target molecules have been synthesized to control and regulate VEGF expression and promote angiogenesis (Zhao et al., 2019). Other bioactive components, such as platelet-rich plasma and exosomes, have also been used for the synthesis of pro-angiogenic hydrogels (Shi et al., 2017; Qian et al., 2020). In addition to delivering pro-angiogenic molecules, vascular networks can be physically engineered to induce blood perfusion from the host tissue to the implant and to promote monocyte function with pro-angiogenic properties (Lee et al., 2020). Thus, hydrogels can be designed to significantly promote angiogenesis in oral and maxillofacial wounds.

### 3.6 Re-epithelialization-promoting hydrogels

Re-epithelialization is an important evaluation index of oral and maxillofacial wound healing and is of great significance for wound closure. Keratinocytes initiate the process of epithelialization, with KGF being an important factor (Ni et al., 2007). However, KGF cannot act directly at the wound site due to its instability. Thus, hydrogel transport of KGF can significantly improve its stability and promote wound re-epithelialization (Wathoni et al., 2019). Epiregulin, a member of the EGF family, is more effective than KGF in promoting wound re-epithelialization. Epiregulin-containing hydrogels can positively promote wound healing by inducing HaCaT cell proliferation and stratification (Reno et al., 2012). In addition to delivering growth factors, the hydrogel delivers oxygen to relieve the hypoxic environment and promote re-epithelialization. Hydrogels containing oxygen-releasing microspheres can promote re-epithelialization of diabetic wounds through continuous oxygen delivery to the wound site (Guan et al., 2021). Therefore, the promotion of re-epithelialization in oral and maxillofacial wounds can be achieved through the use of hydrogels carrying cargos such as KGF, EGF and oxygen.

## 4 Functional drug-loaded hydrogel bio-dressings promote drug release in response to stimulation

**FIGURE 3:**
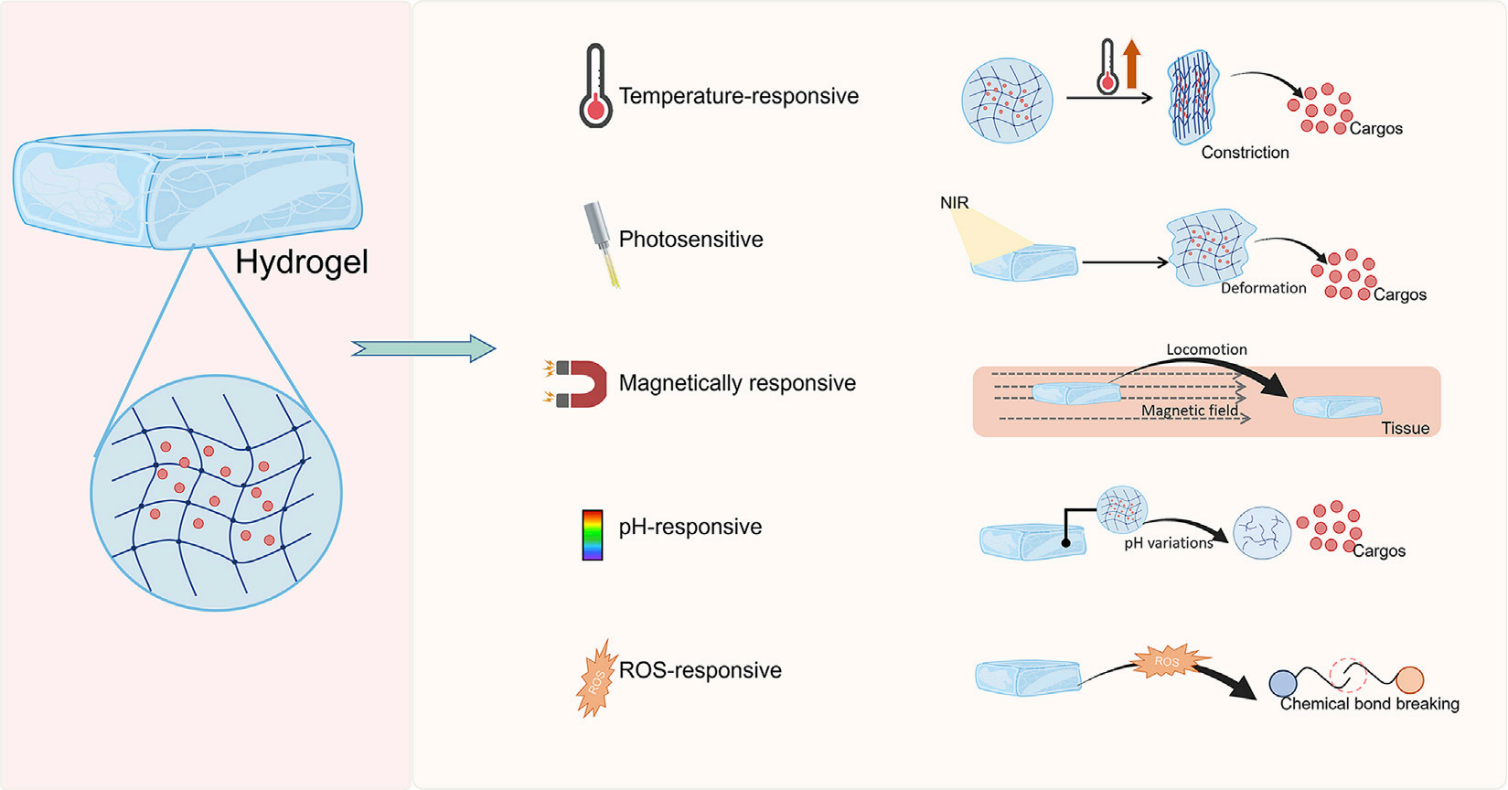
Classification of responsive hydrogels.

The wound healing process involves a variety of cells and growth factors, and conventional dressings cannot regulate the release of active pharmaceutical ingredients in response to the healing process. Smart wound dressings can selectively release active substances according to changes in temperature, light, magnetic fields, pH, enzyme levels, and ROS at the wound site. Furthermore, the synthetic components of hydrogels and the physical characteristics of their 3D pore-like structure allow good drug-loading capacity. Compared to traditional wound dressings, drug-loaded hydrogels can release active substances by responding to the local environment to regulate the different stages of wound healing (Figure 3).

### 4.1 Temperature-responsive hydrogels

Compared with skin, the internal temperature of the oral cavity is relatively high and stable; thus, temperature-sensitive hydrogels can be utilized to achieve responsive release of cargos with temperature variation. Inspired by hot spring shower, hydrogels were prepared from N,O-carboxymethyl CS and fayalite, which can stimulate angiogenesis and promote wound healing by releasing bioactive ions under heat stimulation (Sheng et al., 2021). In addition, thermally responsive hydrogels have been designed to release VEGF in response to body temperature stimulation to promote the healing of diabetic wounds (Chen et al., 2022a). The inflammatory response leads to an increased temperature of wound tissue, and the temperature-responsive properties of the hydrogel allow for the intelligent release of FGF to reduce the inflammatory response (Chen et al., 2018a). Hydrogels have been designed that can change from the gel state to liquid state based on temperature sensitivity, which reduces secondary damage that may be caused during dressing changes (Shi et al., 2022). Therefore, temperature-sensitive hydrogels can achieve drug release according to temperature changes inside the mouth and promote angiogenesis and anti-inflammation, avoiding secondary damage during dressing change.

### 4.2 Photosensitive hydrogels

Some hydrogels can be laser irradiated to disrupt the original structure of the hydrogel and facilitate drug release. Smart hydrogels can respond to light stimulation for drug delivery. Metal ions have photothermal conversion ability, and the release of drugs can be achieved by synthesizing metal ion-loaded hydrogels. Au nanorods functionalized with active peptides have been made into bilayer structured hydrogels to achieve NIR-responsive drug release under NIR irradiation (Huang et al., 2023b). Additionally, given the excellent antibacterial properties of Cu, a multifunctional composite hydrogel was prepared using polydopamine and Cu-doped calcium silicate ceramics. Cu ions are heated by the photothermal effect and produce a unique ‘hot ion effect’, providing antibacterial properties, angiogenesis, and collagen deposition during the healing of infected wounds (Xu et al., 2020). In addition to metal nanoparticles, polydopamine has been used as a photothermal agent for the synthesis of photosensitive hydrogels due to its excellent light absorption in the NIR region; in this way, hydrogels have been developed to release Se nanoparticles (Xu et al., 2023). Therefore, photo-responsive hydrogels can be prepared by designing the components of hydrogels, achieving the responsive release of drugs with antibacterial function to promote wound healing in oral and maxillofacial areas.

### 4.3 Magnetically responsive hydrogels

Homogeneous magnetically responsive nanocomposite hydrogels with enhanced mechanical properties have been obtained by tannin-assisted bridge building between magnetically deformable cobalt ferrite nanoparticles and a polyvinyl alcohol (PVA) matrix. In the presence of an external static magnetic field, the composite hydrogels produced mechanical stimulation-induced early blood vessel formation and accelerated wound healing (Wang et al., 2022b). Additionally, by doping the hydrogel sheet with magnetic nanoparticles, endothelial progenitor cells can be promoted to colonize the wound, enhancing the healing effect of hydrogels (Noh et al., 2019). Curcumin has also been used in the synthesis of magnetic hydrogels, and released curcumin is able to promote tissue repair and regeneration by promoting angiogenesis (Daya et al., 2022). Therefore, angiogenesis can be promoted in oral and maxillofacial wound healing by preparing hydrogels responsive to magnetic fields.

### 4.4 pH-responsive hydrogels

Studies have shown that, when the skin barrier is disrupted, it leads to changes in wound pH, which has implications for the design of pH-responsive wound dressings. A hydrogel synthesized by cross-linking a Schiff base bonds and PF127 micelles is protonated in an acidic environment, leading to breakage of the Schiff base and resulting in the release of curcumin (Qu et al., 2018). Magnesium acetate was also loaded for the synthesis of pH-responsive hydrogels with sustained antibacterial and antioxidant activity (Wang et al., 2022a). In addition to traditional Chinese medicines, studies have shown that exosomes have a positive effect on promoting wound healing. There is a need to develop hydrogel materials that can release exosomes on demand and have exosome compatibility. Hydrogels carrying adipose mesenchymal stem cell exosomes were successfully synthesized by the Schiff base reaction, and the Schiff base bond was broken in the acidic environment of diabetic wounds to release exosomes (Wang et al., 2019a). Therefore, according to the characteristics of pH change in the wound healing process, pH-responsive hydrogels can be designed to achieve cargo release and promote antibacterial, antioxidant, and angiogenesis functions.

### 4.5 ROS-responsive hydrogels

ROS levels in chronic wound sites are high, and ROS-responsive hydrogels can be designed according to these characteristics to achieve intelligent release of drugs and thus improve the therapeutic effect. High glucose in diabetic wounds leads to increased levels of ROS, which increases the duration of the inflammatory response (Brizeno et al., 2016). Synthetic polymers, through the modification of molecular components, can confer specific efficacy to hydrogels. By mixing tannic acid, phenylboronic acid-modified polyphosphonitrile, and PVA, the ROS-responsive anti-inflammatory TA-conjugated nanoparticle hydrogels effectively scavenged ROS, reduced the expression of pro-inflammatory factors, and promoted diabetic wound healing (Ni et al., 2022). PVA-based hydrogels cross-linked with a ROS-responsive linker have also been obtained by modifying the composition of the hydrogels, which gradually degrade due to ROS-responsive cleavage to release loaded mupirocin and the growth factor GM-CSF, which can inhibit bacterial growth (Zhao et al., 2020). ROS-responsive hydrogels can be prepared by designing the components of hydrogels to achieve anti-inflammatory/antioxidant effects on oral and maxillofacial wounds.

### 4.6 Multiple responsiveness hydrogels

During injury to the oral and maxillofacial region, factors such as pH, enzymes levels, and ROS are altered. Reversible covalent bonds in hydrogels are an important feature in designing responsive hydrogels, and many covalent bonds can respond to pH, ROS levels, and temperature. Based on the characteristics of low pH and increased ROS in diabetic wounds, a pH/ROS dual-responsive hydrogel has been designed that releases diclofenac sodium and caffeic acid to inhibit inflammation and promote angiogenesis, achieving rapid healing of diabetic wounds in their acidic oxidative environment (Wu et al., 2022b). In addition, hyaluronic acid has the ability to respond to a variety of external stimuli and has been used to design a pH-responsive (weakly alkaline environment) and hyaluronidase-responsive Zr^4+^-releasing hydrogel that can inhibit bacterial growth and biofilm formation, accelerating wound healing (Zhou et al., 2023a). Keratin is an excellent hydrogel candidate given its multi-stimulus responsiveness to pH and glutathione and enzyme levels (Peralta Ramos et al., 2017; Qiu et al., 2020; Wang et al., 2020). Dual network hydrogels exhibit responsiveness to temperature, pH, and ROS levels as well as excellent antibacterial and antioxidant properties. In particular, these hydrogels respond to both acidic and alkaline environments, and can be used in the treatment of both acute and chronic wounds (Han et al., 2021). The synthetic design of peptide structures imparts excellent thermo-responsive, ultrasound-responsive, and enzymatic-responsive properties to release the anti-inflammatory drug naproxen, which regulates the inflammatory response (Fan et al., 2017). To reduce post-operative complications, including recurrence of residual tumor cells and bacterial infection, a pH, temperature and NIR multi-responsive hydrogel loaded with doxorubicin and indocyanine green has been prepared to kill residual tumor cells on the wound surface and deep in the skin, eliminate harmful bacteria and bacterial biofilms, and promote rapid healing of post-operative tumor wounds (Zhao et al., 2021). In addition, hydrogels can be designed for the treatment of postoperative wounds of oral and maxillofacial tumors (Chen et al., 2022b). Therefore, hydrogels can be designed to be multi-responsive and effective in promoting antibacterial, antioxidant, and angiogenesis properties for a variety of wound types.

## 5 Healing-promoting cargos carried by hydrogels

**FIGURE 4:**
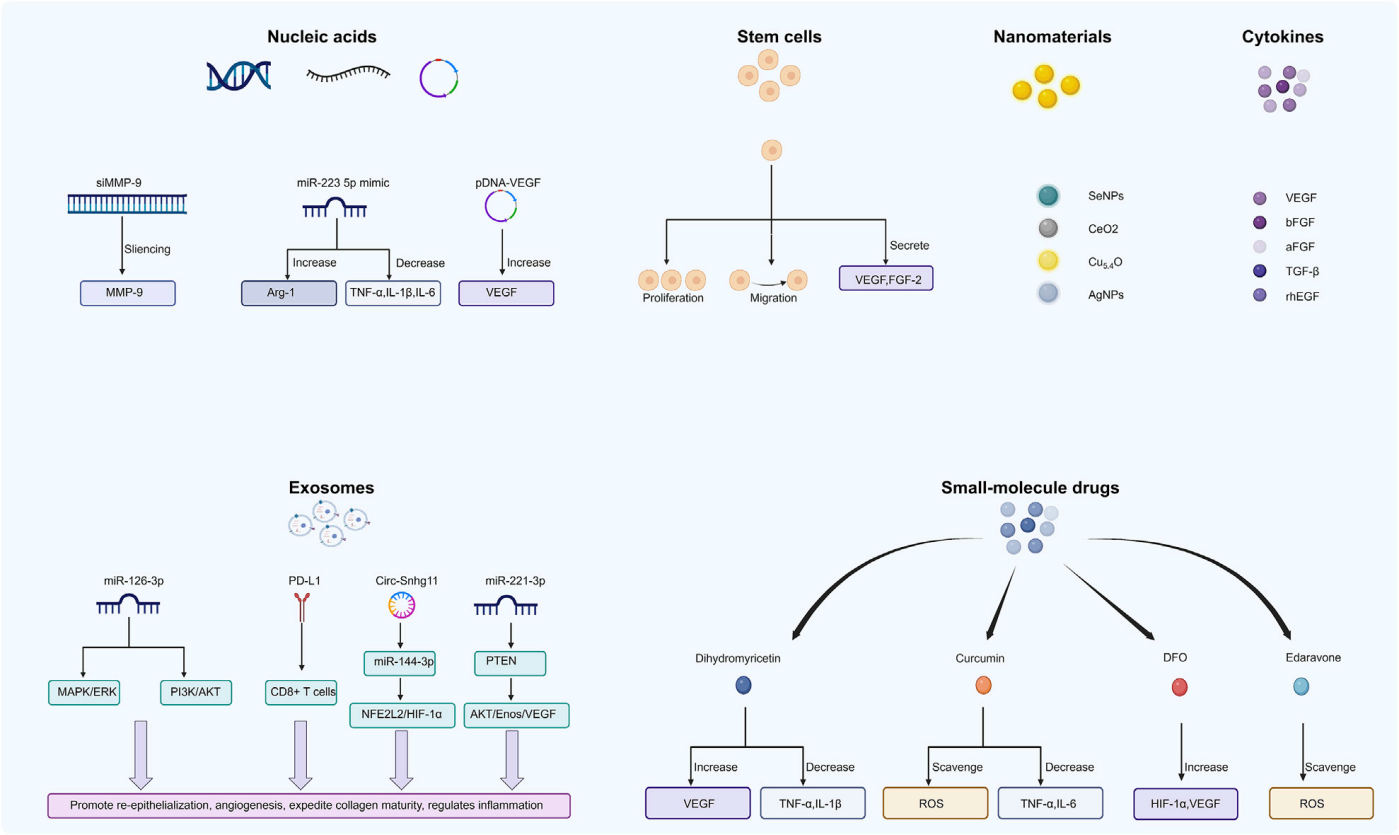
Cargos carried by hydrogels to promote wound healing.

Hydrogels play an important role in promoting wound healing, and their porous structure and biocompatibility allow them to carry a variety of biologically active cargos such as cells and exosomes (Figure 4; Table 2).

**TABLE 2:** Cargos carried by hydrogels to promote wound healing.

Cargos		Function	Wound healing properties of hydrogels	Wound	References
Nucleic acids	MMP-9-targeting siRNA (siMMP-9)	MMP-9 sliencing, reduce the expression of MMP-9.	Increase the collagen content in the granulation tissue and promote the maturation of the granulation tissue.	Diabetic wound	Lan et al. (2021)
	miR-223-5p mimic	Increase expression of Arg-1 and decrease TNF, IL-1β, and IL-6.	Promote the polarization of M2 type macrophages, reduce inflammatory response, and promotes the formation of vascularized skin tissue.	Wound	Saleh et al. (2019)
	pDNA-VEGF	Promote VEGF expression.	Inhibit inflammation and promote angiogenesis.	Burn wound	Wang et al. (2019a)
	PDRN	Improve angiogenesis, promote cellular activity, increase collagen synthesis, and exert anti-inflammatory effects.	Promote angiogenesis, cell proliferation, and collagen synthesis.	Wound	Jing et al. (2021)
	PDRN	Promote tissue regeneration and anti-inflammation.	Promote proliferation and migration of epidermal fibroblasts and increase collagen synthesis.	Wound	Sun et al. (2023a)
	miRNA-146a	Reduce expression of pro-inflammatory genes and promote Col1a2 gene expression.	Reduce inflammation and improve wound healing.	Diabetic wound	Sener et al. (2020)
Cytokines	VEGF	Promote angiogenesis.	Enhanced anti-inflammatory response, angiogenesis and cell proliferation at the wound site to promote rapid wound healing.	Wound	Siebert et al. (2021)
	aFGF, bFGF	Promote cell proliferation, granulation formation, re-epithelization, and angiogenesis.	Promote granulation formation, re-epithelization, and angiogenesis.	Wound	Wu et al. (2016)
	TGFβ-inhibitor	Regulate cell proliferation, migration, ECM production and immune response.	Enhance wound closure and enhance scarless wound repair.	Wound	Zhang et al. (2021a)
	rhEGF	Promote cell proliferation.	Promotes fibroblast proliferation and accelerates diabetic wound closure.	Diabetic wound	Hajimiri et al. (2016)
Small-molecule drugs	Curcumin	Scavenge excess ROS and reduce the expression of pro-inflammatory cytokines (IL-6 and TNF-α).	Reduce the inflammatory response and promote angiogenesis.	Burn wound	Yang et al. (2021)
	Dihydromyricetin	Antioxidant, antibacterial, anti-inflammatory.	Promote the expression of skin-related growth factors, angiogenesis and collagen production and inhibit inflammation.	Wound	Zhao et al. (2022)
	Edaravone	Scavenge excess ROS.	Reduce oxidative stress at the wound site and promote diabetic wound healing.	Diabetic wound	Fan et al. (2019)
	DFO	Regulate HIF-1α.	Promote angiogenesis and reduce inflammation in diabetic wounds, and antibacterial.	Diabetic ulcers	Li et al. (2022a)
	Resveratrol	Anti-inflammatory, antioxidant, antibacterial and anti-aging.	Anti-inflammatory, antioxidant, antibacterial.	Wound	Jia et al. (2022)
	Baicalin	Antioxidant and scavenge excess ROS.	Regulate inflammatory response and promote wound healing.	Wound	Manconi et al. (2018)
Stem cells	Adipose stem cells	Secrete multiple pro-angiogenic factors.	Promote angiogenesis.	Wound	Eke et al. (2017)
	Human adipose stem cells	Promote cell proliferation and regulate inflammatory response.	Promote re-epithelialization, nerve fiber growth and modulate the inflammatory response.	Diabetic ulcers	da Silva et al. (2017)
	Bone marrow mesenchymal stem cells	Promote growth factor secretion and regulate macrophage polarization.	Inhibit inflammation and promote angiogenesis.	Diabetic ulcers	Bai et al. (2020)
	Mesenchymal Stem Cell Spheroids	Angiogenesis, anti-inflammatory, paracrine effects.	Accelerate epithelial remodeling and angiogenesis.	Diabetic wound	Yang et al. (2020b)
Exosomes	Mesenchymal stem cell-derived exosomes	Vascular endothelial growth factor (VEGF) and factor transforming growth factor beta-1 (TGFβ-1) expression are upregulated.	Promote angiogenesis.	Diabetic wound	Yang et al. (2020a)
	hypoxia-pretreated ADSC-Exo	Circ-Snhg11 activates miR-144-3p/NFE2L2/HIF1α signaling.	Promote angiogenesis.	Diabetic wound	Hu et al. (2023)
	ATV pretreated BMSC derived exosomes	MiR-221-3p activates the AKT/eNOS pathway and promotes endothelial cell proliferation, migration, tube formation and VEGF levels.	Promote angiogenesis.	Diabetic wound	Yu et al. (2020)
	Exosomes with high concentration of PD-L1	Inhibit T-cell activation and promote migration of epidermal cells and dermal fibroblasts.	Regulate immune response, accelerate wound contraction and re-epithelialization.	Wound	Su et al. (2019)
Nanomaterials	Selenium nanoparticles	Antioxidant and anti-inflammatory.	Reduce inflammation and promote wound closure, granulation tissue formation, collagen deposition, angiogenesis, fibroblast activation and differentiation.	Wound	Mao et al. (2021)
	Cerium oxide nanoparticles	Antibacterial, antioxidant, anti-inflammatory and anti-cancer.	Induce collagen deposition and accelerate wound closure.	Wound	Ahmed et al. (2021)
	nanoenzyme-Cu_5.4_O	Scavenge ROS.	Reduce inflammation, increase epidermal regeneration, angiogenesis.	Wound	Peng et al. (2021)
	Silver nanoparticles	Antibacterial and antioxidant.	Promotes diabetic wound healing through antibacterial, accelerated collagen deposition and re-epithelialization.	Diabetic wound	Masood et al. (2019)

### 5.1 Nucleic acids

The process of wound healing is regulated by a variety of nucleic acids, and therefore the regulation of wound healing can be achieved through the application of nucleic acid-based drugs. MMPs are involved in tissue remodeling; however, increased levels of MMP9 expression in chronic non-healing wounds lead to local ECM breakdown and delay the wound healing process. Small interfering RNAs can effectively silence gene expression and are highly promising for wound healing applications. Sustained local delivery of MMP9-targeting small interfering RNA (siMMP9) has been achieved through hydrogel loading, which significantly improved diabetic wound closure by silencing MMP9 (Lan et al., 2021). MicroRNAs are capable of targeting multiple genes in specific biological processes to produce lasting effects on disease treatment at the molecular level. Hydrogels have been designed and synthesized loaded with a miR-223-5p mimic, which promotes the polarization of macrophages to M2-type macrophages in wound tissue and upregulates the expression of the anti-inflammatory gene *Arg-1* and downregulates the expression of TNF, IL-1β, and IL-6 (Saleh et al., 2019). DNA plasmids have low transfection efficiency, while their transport with hydrogels can improve their delivery efficiency. *VEGF* gene transfection was promoted by delivering plasmid DNA encoded with VEGF through hydrogels, inhibiting the inflammatory response and promoting microvessel formation (Wang et al., 2019b). In addition, polydeoxyribonucleotides (PDRN) also have a healing-promoting effect, and wound healing has been shown to be significantly accelerated by the synthesis of polysaccharide-based hydrogels containing PDRNcarriers that release the gene carrier PCNP for effective uptake by skin fibroblasts (Jing et al., 2021). Therefore, the hydrogel can be used to carry nucleic acids and improve their stability and utilization, with application in the treatment of oral and maxillofacial wound healing through regulation of the inflammatory response and promotion of angiogenesis.

### 5.2 Cytokines

Cytokines at the wound site play an important regulatory role in the healing process; therefore, the topical delivery of cytokines, such as VEGF, basic FGF (bFGF), TGFβ, and rhEGF, through hydrogels has a significant effect on wound healing (Wu et al., 2016). A 3D-printed hydrogel patch coated with VEGF and modified by tetrapodal zinc oxide particles has been developed to achieve controlled release of VEGF and regulate angiogenesis during wound healing (Siebert et al., 2021). bFGF is involved in the physiological process of wound healing and has strong biological activity. A hydrogel system with continuous delivery of bFGF was prepared with a significant effect on wound healing in aged rats (Xiao et al., 2022). A microencapsulated hydrogel system that releases TGFβ inhibitor has been prepared to achieve temporal specific management of skin wounds and to promote scar-free healing (Zhang et al., 2021a). A study has also modified the growth factor and prepared carboxymethyl CS recombinant human epidermal growth factor coupling (NaCMCh-rhEGF), which can protect rhEGF from degradation by protease and improve the application of the drug (Hajimiri et al., 2016). The cytokine-loaded hydrogel can be used for oral and maxillofacial wound healing by reducing the inflammatory response and promoting angiogenesis.

### 5.3 Small-molecule drugs

A variety of small-molecule drugs have been developed for anti-inflammatory, antioxidant, and angiogenesis purposes. Drug-delivery hydrogels can improve the utilization rate, stability, and sustained release of drugs to improve their therapeutic effect, whilst reducing their toxic side effects. By loading curcumin into a hydrogel, an improved capacity to reduce ROS and regulate the inflammatory response can be achieved (Yang et al., 2021). Dihydromyricetin, a natural polyhydroxyflavonoid with antioxidant, antibacterial, and anti-inflammatory properties, has been shown to promote skin repair when loaded in a temperature-sensitive hydrogel by promoting the expression of skin-related growth factors and inhibiting the expression of cellular inflammatory factors (Zhao et al., 2022). Diabetic wounds are difficult to heal due to impaired angiogenesis, and the increased inflammatory response caused by excessive ROS in wounds is a major factor affecting healing. Edaravone can effectively remove ROS but its restricted stability limits its application. Loading edaravone onto hydrogels can improve its stability and drug utilization and promote the rapid healing of diabetic wounds through slow release (Fan et al., 2019). DFO is an FDA-approved small-molecule drug that accelerates the healing of diabetic ulcers by regulating VEGF and HIF1α in a long-lasting and stable manner through hydrogel-carrying DFO (Li et al., 2022b). Resveratrol and baicalin have also been used in drug-loaded hydrogels to promote wound healing (Manconi et al., 2018; Jia et al., 2022). Thus, hydrogels can carry various small-molecule drugs to achieve oral and maxillofacial wound healing by promoting antibacterial, anti-inflammatory/antioxidant, and angiogenesis properties.

### 5.4 Stem cells

Stem cells can produce and secrete growth factors that stimulate angiogenesis and re-epithelialization (Kim et al., 2009). Moreover, hydrogels can provide an excellent storage environment for stem cells. Studies have shown that the angiogenic effect of hydrogels is increased threefold after the addition of adipose stem cells, which can significantly promote wound healing (Eke et al., 2017; Shiekh et al., 2020). Human umbilical cord mesenchymal stem cells encapsulated in hydrogels can promote angiogenesis and collagen deposition by inhibiting the secretion of inflammatory factors TNF and IL-1β, with accelerated healing effects on diabetic wounds (Xu et al., 2022). Embryonic stem cells, induced pluripotent stem cells, and mesenchymal stem cells have shown good efficacy in promoting wound healing in hydrogel-stem cell therapy by inhibiting inflammation and promoting angiogenesis (Li et al., 2022a; Huang et al., 2022). Thus, hydrogels loaded with stem cells may promote oral and maxillofacial wound healing by inhibiting inflammation and promoting angiogenesis.

### 5.5 Exosomes

Exosomes are small vesicles secreted by cells that carry a variety of nucleic acids and proteins. The addition of exosomes to hydrogels has shown an excellent effect on the healing of diabetic wounds, a reduction in the formation of scar tissue, and promotion of the production of skin appendages, with a positive effect on skin regeneration (Wang et al., 2019a). Exosomes, as bioactive substances, have modifiable properties. By preparing exosomes derived from miR-126-3p overexpressed synovial mesenchymal stem cells, the exosomes obtain re-epithelialization, angiogenesis, and collagen maturation effects, promoting diabetic wound healing (Li et al., 2016). Moreover, as a product of cells, exosomes retain the ability to regulate wound healing and immune tolerance and are more readily taken up by recipient cells; therefore, exosome-loaded hydrogels have the potential to promote wound healing (Safari et al., 2022). Exosomes can be engineered and loaded to promote oral and maxillofacial wound healing by regulating the wound inflammatory microenvironment, promoting angiogenesis, and enhancing re-epithelialization (Su et al., 2019; Yu et al., 2020; Hu et al., 2023).

### 5.6 Nanomaterials

Metal nanomaterials with antioxidant, antibacterial, and wound-healing properties have also been used in hydrogels (Hu et al., 2017). Metal nanoenzymes have a large number of catalytically active surface atoms and are therefore highly active and more stable than natural enzymes (Yadi et al., 2018). For example, selenium nanoparticles have significant antibacterial, anti-inflammatory, and antioxidant effects (Mao et al., 2021). The addition of cerium oxide nanoparticles to a CS hydrogel provides antioxidant and antibacterial properties, significantly improving the wound healing process (Ahmed et al., 2021). In addition, a composite hydrogel loaded with ultra-small nanoenzyme–Cu_5.4_O can chelate inflammatory chemokines and inhibit inflammatory cell activity, with the continuous release of Cu_5.4_O promoting angiogenesis (Peng et al., 2021). Most metal nanoparticles have antibacterial effects, especially silver nanoparticles; therefore, the addition of silver nanoparticles into hydrogels and their slow release lead to a continuous antibacterial effect (Masood et al., 2019). The healing of oral and maxillofacial wounds can thus be promoted by hydrogels loaded with nanomaterials through antibacterial, anti-inflammatory, and antioxidant effects.

## 6 Discussion and conclusion

The skin and mucous membranes of the oral and maxillofacial region are located in exposed areas of the body and are susceptible to interference from internal and external environmental factors, resulting in destruction of the epidermal barrier and the formation of wounds. In addition, there are structural biological differences between the oral mucosa and skin (Nikoloudaki et al., 2020). Both oral mucosa and skin are composed of an epithelial layer and connective tissue but part of the oral mucosal epithelium does not contain a keratinized layer (Qin et al., 2017). Moreover, the oral mucosa is in a more humid, friction-prone, and bacteria-rich environment than the skin and has no skin appendages (Glim et al., 2013). Compared to skin, oral mucosa requires dressings and medications with good adhesion in wet environments as well as antimicrobial properties, friction resistance, and anti-inflammatory properties (Table 3). Thus, considering the differences between skin and oral mucosa, functional and suitable hydrogels should provide varying effects of hemostasis, inflammation, proliferation, and remodeling for wound healing.

**TABLE 3:** Characteristics and effects of hydrogels in oral wounds.

Oral wound	The characteristics of hydrogels	Effects of application of hydrogels	References
Humid and highly dynamic oral wound environments.	Photocrosslinking, adhesion and degradability.	The hydrogel adheres to the oral mucosa, protects mucosal wounds from liquid flushing, oral movements and friction, and promotes healing of oral mucosal injuries in rats and pigs.	Zhang et al. (2021b)
Tooth-Extraction Wound Healing.	High strength, quick gel formation, injectable, anti-inflammatory.	The hydrogel is able to rapidly form a gel within seconds, has excellent antimicrobial properties, and is able to reduce the inflammatory infiltration of rat tooth extraction wounds.	Wang et al. (2022)
Moist and dynamic oral wound environment.	Protects the wound and isolates it from the external environment, with good resistance to dissolution, adhesion and adherence.	The hydrogel cross-links *in situ* to protect the wound, reduce acute inflammation in canine extraction wounds, and promote rapid healing of intraoral wounds.	Wu et al. (2022b)
Moist and dynamic oral wound environment.	Favorable adhesion properties, anti-oxidation.	The hydrogel was able to adhere to moist oral mucosal wounds and possessed excellent antioxidant ability, which could effectively shorten the inflammation period and promote the healing of oral wounds in diabetic rats.	Sun et al. (2023)
Dark, persistent bacterial irritation of oral mucosal wounds	Antibacterial.	The hydrogel possesses favorable antimicrobial ability and can promote the rapid healing of oral mucosal wounds in rats.	Qi et al. (2023)
Moist and dynamic oral wound environment.	Hemostatic and antimicrobial.	The hydrogel reduces inflammatory reactions, bleeding, and antimicrobial activity, thereby promoting rapid wound healing in patients with oral mucosal injuries.	de Jesus et al. (2023)
Salivary gland excision wound.	Promotes salivary gland regeneration.	The hydrogel sheet was able to promote the regeneration of salivary glands at the wound of submandibular gland excision in rats.	Miyake et al. (2020)
Periodontal damage.	Antibacterial, osteogenic.	The hydrogel resists *Porphyromonas gingivalis*, promotes osteogenesis and accelerates periodontal wound healing.	Zang et al. (2019)
Bacterial irritation.	Antibacterial, protects wounds.	The hydrogel is antimicrobial but does not affect the oral commensal microbiota and accelerates healing of infected extraction wounds.	Chen et al. (2023)
Direct exposure to the external environment.	Regulating macrophages.	The hydrogel modulates macrophage differentiation, inhibits transitional inflammatory responses, and promotes rapid repair of oral mucosal damage.	Wen et al. (2023)

Traditional dressings are not able to fully meet the needs of oral and maxillofacial wound treatment. Thus, addressing wound healing in the oral and maxillofacial region is urgently required. Hydrogels have shown promising application in wound healing of the oral and maxillofacial region (Zhou et al., 2023b). Hydrogels can accelerate the healing of many types of wounds, including chronic difficult-to-heal wounds such as diabetic wounds, inflammation, and burns, through good adhesion, anti-inflammatory/antioxidant effects, antibacterial effects, hemostasis, promotion of angiogenesis, and re-epithelialization (Li et al., 2022a). The oral mucosa is moist and the environment with saliva and food flushing requires the hydrogel to have excellent tissue adhesion ability and be able to maintain a long adhesion time on tissues. In addition, the oral mucosa is in direct contact with the external environment and is prone to inflammation and oxidation, and prolonged inflammation that is difficult to suppress may lead to tissue necrosis and reduced function of the oral and maxillofacial area (Senel, 2021; Ngeow et al., 2022). The anti-inflammatory and antioxidant properties of hydrogels help to alleviate oxidative stress and reduce the inflammatory response (Luneva et al., 2022). Bacteria in the oral cavity can become pathogenic when the body is compromised or immunocompromised, causing slow wound healing. Hydrogels can prevent bacterial colonization and inhibit bacterial growth. Therefore, hydrogels should have adhesive, anti-inflammatory/antioxidant, and antibacterial properties. In the complex oral environment, hydrogels adhere to moist wounds and protect them from the external environment without destroying oral microflora (Zhang et al., 2021b; Chen et al., 2023). For periodontal, extraction and salivary gland tissues, hydrogels promote periodontal repair, bone regeneration, and salivary gland regeneration as well as resisting bacterial infection, which has a positive effect on the restoration of oral tissue structure and function (Zang et al., 2019; Miyake et al., 2020).

Further, functional hydrogels have the ability to respond to magnetic fields, light, temperature, and pH changes in wound healing. For example, given that oral and maxillofacial wounds are located on the surface of the body, an applied magnetic field and light can directly modulate hydrogel release without additional damage to the tissue. The temperature inside the oral cavity is relatively constant at ∼37°C, such that the temperature-sensitive hydrogel can be designed to apply to the oral cavity (Sund-Levander et al., 2002). In addition, the temperature of the wound increases during inflammation, and the drug can be released specifically in response to this change (Zhou et al., 2019). Thus, responsive hydrogels can be designed to respond to the particular internal environmental changes of wounds, such as pH, enzymes, peroxide environment, high ROS levels, and high glucose levels, thus achieving on-demand release of the drug-loaded hydrogel. The healing process of oral and maxillofacial trauma is complex. Most smart hydrogels only regulate one of these stages, and hydrogels should be designed that can track and regulate all of the different healing stages (Huang et al., 2023).

Changes in temperature, light, magnetic field, pH, and ROS in functional hydrogels can also be exploited to achieve effective cargo release where required. Indeed, the effects of hemostasis, inflammation, proliferation, and remodeling of oral and maxillofacial wound healing depend on the delivery of cargos such as RNAs, growth factors, drugs, stem cells, and exosomes (McCarthy et al., 2021; Teixeira et al., 2021; Qi et al., 2023). Previous reports indicated that miR-31, miR-21, miR-23b, miR-200 and VEGF, EGF, HGF growth factors promote cell proliferation and influence inflammation in both oral mucosa and skin (Nagy et al., 2001; Chen et al., 2019; Simoes et al., 2019). Furthermore, evidence has suggested that functional stem cell-loaded and exosome-loaded hydrogels significantly promote wound healing through MAPK, Wnt, HIF-1, IGF1R/AKT/mTOR, and TGFβ signaling pathways in oral and skin wounds (Prime et al., 2004; Eslami et al., 2009; Zhang et al., 2009; Steenhuis et al., 2011; Calenic et al., 2015; Houschyar et al., 2020; Zhou et al., 2022).

Oral and maxillofacial wound healing undergoes hemostasis, inflammation, proliferation, and remodeling and can be improved by functional and responsive hydrogels that can be applied to oral wounds and promote wound healing.
